# A Pilot Field Evaluation of Dietary Ginger *Zingiber officinale* Effects on Immunity, Blood Metabolic Profile, and Disease Resistance in *Labeo rohita* Under Semi-Intensive Farming

**DOI:** 10.3390/biology14020135

**Published:** 2025-01-28

**Authors:** Priya Rawat, Vaneet Inder Kaur, Anuj Tyagi, Parisa Norouzitallab, Kartik Baruah

**Affiliations:** 1 Department of Aquaculture, College of Fisheries, Guru Angad Dev Veterinary and Animal Sciences University (GADVASU), Ludhiana 141004, PB, Indiavaneetinderkaur@gadvasu.in (V.I.K.); 2 Department of Aquatic Environment, College of Fisheries, Guru Angad Dev Veterinary and Animal Sciences University (GADVASU), Ludhiana 141004, PB, India; anujtyagi@gadvasu.in; 3 Department of Applied Animal Science and Welfare, Faculty of Veterinary Medicine and Animal Sciences, Swedish University of Agricultural Sciences, 75007 Uppsala, Sweden; parisa.norouzitallab@slu.se

**Keywords:** *Aeromonas hydrophila*, disease resistance, ginger powder, immune responses, *Labeo rohita*

## Abstract

A feeding trial was conducted for 120 days in a pilot field condition to evaluate ginger powder (GP; *Zingiber officinale*) as a feed supplement for *Labeo rohita* to improve immunity, blood metabolic profile, and resistance to *Aeromonas hydrophila* bacterial infection. The study was conducted following a complete randomized design in outdoor cemented tanks (20 m^2^) with a stocking density of 30 fish (average weight: 20.5 g) per tank. *L. rohita* fingerlings were divided into five groups and fed a diet with no GP supplementation (control diet; C) or a diet supplemented with 5 g (GP5), 10 g (GP10), 15 g (GP15), or 20 g (GP20) of GP per kg of the control diet. The results showed that fish well-being, determined by measuring the condition factor, remained higher than 1.0 for all the experimental groups. The condition factors of all the groups were not significantly different between the groups, suggesting GP had no negative impact on overall fish health. Immune responses significantly improved in the groups fed with diets supplemented with GP at doses of 10 to 15 g per kg of their diet, as seen in respiratory burst activity, total immunoglobulins, and lysozyme activity at 60 and 120 days. Blood metabolic variables, particularly the albumin level, tended to show some level of improvement in the GP15-fed group. However, the effect was not significant. Total protein and globulin levels and the albumin/globulin ratio were not significantly affected by dietary GP. Importantly, fish fed the GP15 diet showed the highest resistance to the *A. hydrophila* challenge. In conclusion, ginger powder holds promise as a health-promoting nutraceutical for farmed *L. rohita*, contributing to their sustainable farming.

## Simple Summary

This study is a validation trial that aims to verify the positive effects of feeding dietary ginger powder on the defense responses and disease resistance in *Labeo rohita*, a widely farmed carp in Southeast Asia. The trial was conducted in a pilot field setting under semi-intensive farming conditions over 120 days. The fish were fed either a control diet or diets supplemented with varying levels of ginger powder (GP): 5 g (GP5), 10 g (GP10), 15 g (GP15), or 20 g (GP20) of GP per kg of the control diet. The results showed a marked enhancement in the fish’s immune response when dietary GP was fed. This improved immunity was linked to increased resistance to bacterial diseases caused by *Aeromonas hydrophila*. Thus, ginger can be used as a natural additive instead of chemotherapeutics, such as antibiotics, to enhance the robustness of the fish in support of sustainable farming.

## 1. Introduction

Freshwater aquaculture is a vital source of protein globally. In South Asian countries, carp are the primary species cultured in freshwater, making up around 85% of the total freshwater production, such as in India [1]. They contribute significantly to the economy and nutritional security. The Indian major carp *Labeo rohita,* commonly known as rohu, is highly preferred as a food species in the Indian subcontinent. Its total production is 2.48 million metric tons [1]. Disease caused by the bacterial pathogen *Aeromonas hydrophila* remains one of the major problems for the sustainable farming of carp that include *L. rohita* [2]. *A. hydrophila* is a Gram-negative, facultative anaerobe bacterium that is motile and opportunistic. It can cause diseases in carp when there are predisposing stressors, subsequently leading to significant economic losses [2]. As a measure to control bacterial infection, farmers use chemotherapeutics, such as antibiotics and disinfectants, in the aquaculture environment. The indiscriminate use of such synthetic compounds can lead to the emergence of antimicrobial resistance strains, posing a threat to the health of farmed (aquatic) animals and humans globally [3]. To control diseases in farmed fishes without the use of unsustainable chemotherapeutics, increasing attention has been paid over the past few years to utilizing naturally occurring botanicals that have the potential to be used as alternatives to chemotherapeutics [4]. The use of botanicals in aquaculture animals is gaining interest due to several reasons: (i) Botanicals are often considered more environmentally friendly, reducing the risk of pollution and harm to ecosystems. (ii) Botanicals often contain bioactive compounds with multifunctional properties that include immune-stimulation activities, enhancing the defense responses of aquaculture species [5]. This, in turn, improves their overall health and disease resistance without the potential negative consequences of chemotherapeutics. (iii) They are easy to access and cost-effective to use as supplements in the diet of farmed fish [6].

Ginger (*Zingiber officinale*), a creeping perennial rhizome native to Asia, including India, belongs to the Zingiberaceae family [7]. It is one of the most important and extensively utilized herbs globally, owing to its wide variety of health-beneficial properties, such as immune stimulation, appetite enhancement, and growth promotion resulting from enhanced activity of digestive enzymes. Ginger is composed of several bioactive components, such as gingerols, paradols, shogaols, and zingerone. The fresh rhizome contains gingerols and zingibain, which are the main active components in the fresh rhizome, known for their anti-inflammatory, anti-microbial, and immune-enhancing effects. These have improved the growth and welfare of animals [7,8,9]. According to a study conducted by Talpur et al. [10], feeding ginger to Asian seabass had a positive impact on their immune system. This led to an increase in the resistance of the fish towards a challenge with the pathogenic bacteria *Vibrio harveyi*. These findings were complemented by other studies in rainbow trout [11] and Mozambique tilapia [12] that showed that feeding ginger extract at 1% markedly improved both humoral (lysozyme activity, total protein, and globulin levels) and cellular (phagocytic activity, respiratory burst activities) immune responses. Moreover, feeding Mozambique tilapia with ginger extract increased their survival rate during a challenge with *V. vulnificus*. In a previous study conducted in a laboratory setting, Sukumaran et al. [13] investigated the impact of feeding dietary ginger for a period of 60 days on the growth and immune responses of the fish and its susceptibility towards *A. hydrophila* infection. The results showed that dietary ginger, when included at a 0.8% level, significantly improved the growth of the fish compared to the control group. The same group exhibited an increase in the skin mucosal immune responses, which were measured in terms of lysozymes, bacterial growth inhibition activities, the level of immunoglobulin and total protein, as well as immune and antioxidant defense-related molecules. The improved immune responses were positively associated with increased resistance of the fish towards *A. hydrophila* infection [13]. The current study was built upon the findings of Sukumaran et al. [13]. In our current study, we aimed to validate the findings obtained from the laboratory study [13] in pilot field conditions following a long-term feeding trial under a semi-intensive farming practice, which is a commonly used farming practice for culturing carp in India and neighboring countries. Here, we present findings suggesting that dietary GP could confer successful protection to *L. rohita* against an *A. hydrophila* challenge and that the protection by GP was associated with the induction of the defense response of the fish.

## 2. Materials and Methods

### 2.1. Experimental Animal and Maintenance

**Figure 1:**
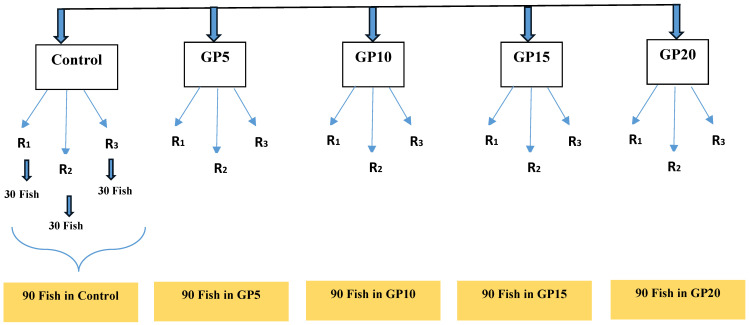
Experimental design depicting the number of replicates and the experimental fish. Four hundred and fifty fingerlings (average initial weight: 20.5 g) were randomly divided into five experimental groups. One group, represented as the control, was fed with a control diet for 120 days. The other four groups were fed a diet supplemented with different inclusion levels of GP: 5 g (GP5), 10 g (GP10), 15 g (GP15), and 20 g (GP20) per kg of the control diet. Each group was maintained in three replicates (indicated as R1, R2, and R3) outdoors in cemented experimental tanks (20 m^2^) with a capacity of 20,000 L of water. Each replicate contained 30 fish.

A total of 550 *Labeo rohita* fingerlings were used in this study. They were raised at the fish farm located at the College of Fisheries, Guru Angad Dev Veterinary and Animal Sciences University, Punjab, India. Before the start of the feeding experiment, the fingerlings were acclimatized to the experimental conditions in 500-L fiberglass tanks for 15 days. During this period, the fish were fed a control diet of 3% of their body weight. After acclimatization, a group of 450 fingerlings (n = 450; initial mean weight: 20.5 g) were collected and distributed randomly into five experimental groups with 90 fish per group. Each group was kept in triplicate in outdoor cemented experimental tanks (20 m^2^) with 20,000 L of water capacity (see Figure 1 for experimental design). The tanks were lined with 1–2 inch soil to maintain natural environmental conditions, and lime was applied at the start of the experiment to disinfect and balance the pH at 300 kg/ha. The batch culture system was followed in this study. Water samples were collected every two weeks during the experimental period, in the morning hours, to analyze water quality parameters using standard protocols [14]. The physicochemical parameters of the rearing water analyzed included temperature, pH, dissolved oxygen, total alkalinity, total hardness, and ammonia nitrogen. The temperature (26.0–29.1 °C), pH (7.2–8.6), dissolved oxygen (6.0–11.0 mg L^−1^), total alkalinity (178.7–249.3 mg L^−1^), total hardness (180.0–250.7 mg l^−1^), and ammonia (0.009–0.09 mg l^−1^) remained within the optimal range for carp culture throughout the experimental period [15].

### 2.2. Experimental Diets and Design

Fresh ginger was obtained from a local market in Ludhiana District, Punjab, India. Ginger powder (GP) was prepared following our previously described procedures [16]. Five different experimental diets were developed by mixing GP with several previously ground feed ingredients at different inclusion levels. One diet served as a control, which did not contain GP. The other four test diets contained GP at different levels of inclusion: 5 (GP5), 10 (GP10), 15 (GP15), and 20 (GP20) grams per kg of the control diet. The ingredients were mixed thoroughly and evenly. Water was added to the mixture to produce feed pellets using a hand pelletizer. The resulting pellets of 2 mm size were dried at 60 °C for 24 h and then sealed in air-tight bags for later use. The diets were analyzed for proximate composition in three replicates, according to AOAC’s official methods of analysis [16]. The data on the proximate composition of the feed ingredients and the experimental diets are previously published [16]. The fish in each group were fed twice a day at 10:00 and 16:00 h with ginger-supplemented diets at 5% of the body weight for the initial 60 days. After that, the feeding rate was reduced to 3% until day 120 [16]. Three replicates were maintained for each group. The amount of feed provided was adjusted after each sampling based on the increase in the fish weight following the management strategy, as previously described [17,18]. This approach helps prevent issues related to overfeeding, thereby reducing feed waste and maintaining water quality. Additionally, approximately 5–10% of the water in the experimental tanks was replaced with fresh water every two weeks to compensate for evaporation loss and maintain stable water parameters. The water used in this experiment was sourced from a reservoir tank that was pretreated with limestone.

### 2.3. Experimental Analyses

#### 2.3.1. Condition Factor

After the 120-day feeding trial, we determined the condition factor using the formula previously described [19].

Condition factor = Body weight (g)/Body Length (cm)^3^ × 100

#### 2.3.2. Blood and Serum Collection

At day 60 and the end of the feeding trial (day 120), 6 fish from each replicate tank, with a total of 18 fish from each experimental group, were randomly sampled. They were anesthetized using a mixture of clove oil and ethanol at 1:9 proportion. The clove oil concentration was 50 mg L^−1^. Blood samples were drawn from the caudal vein using a sterile disposable syringe that was prerinsed with heparin. These samples were used for respiratory burst activity assay. To obtain the serum, blood was drawn without heparin and transferred into an Eppendorf tube following the method previously described [20]. The tube was kept at room temperature for 6 h, after which it was centrifuged for 10 min at 4500 g. The supernatant was then carefully separated and stored at a temperature of −20 °C to perform immunological analysis later.

##### Respiratory Burst Activity

The nitroblue tetrazolium assay was used to measure respiratory burst activity from the day 60 and 120 samples [21]. In brief, a blood sample (100 µL) from each experimental group was mixed with 100 µL of 0.2% nitroblue tetrazolium in equal proportion and incubated for 30 min at 25 °C. After incubation, 50 µL of the solution was taken out and mixed with 1 mL of N, N-dimethylformamide in the Eppendorf. The mixture was then centrifuged at 300× *g* for 5 min. The supernatant was measured at 540 nm using a microplate reader (Infinite M200 PRO, Tecan, Männedorf, Switzerland) to determine the optical density.

##### Total Immunoglobulin Level

The total immunoglobulin (Ig) level in the serum sampled on days 60 and 120 was measured, following the method described by Anderson and Siwicki [21]. Briefly, 100 µL of the serum was mixed with an equivalent volume of 12% polyethylene glycol. The solution was incubated at room temperature for 2 h with constant shaking, which was followed by centrifugation at 2200× *g* for 10 min. The supernatant was then taken out, and the protein concentration was determined. The resulting protein reading (or protein concentration) was used to calculate the total immunoglobulin using the following formula:

Total Immunoglobulin level (g dL^−1^) = total protein in the individual serum sample − total protein taken out by absorption to polyethylene glycol.

##### Lysozyme Activity

The turbidimetric test, with some modifications, was used to determine the lysozyme activity in the serum collected on days 60 and 120, as previously described [22]. The serum (50 μL) was mixed with 50 μL of PBS (10 mM, pH 5.8) in triplicate in a 96-well plate. The serum was then serially diluted until the last well, from which 50 μL of the sample was discarded. Next, a fresh culture of *Micrococcus luteus*, grown for 24 h, was centrifuged, and the pelleted cells were washed twice with PBS (10 mM, pH 7.4). The concentration of cells was adjusted to 0.5–0.7 using a spectrophotometer at 450 nm. Then, 125 μL of *M. luteus* was added to each well. The absorbance at 450 nm was measured using an ELISA microplate reader at room temperature for 5 min with 30-s intervals. To prepare a standard curve, hen egg white lysozyme was used.

#### 2.3.3. Blood Metabolic Profile

The blood serum sampled at the end of the 120-day feeding trial was analyzed for total protein and albumin following the method of Gornall et al. [23] by using Erba Kits, Erba Mannheim (Germany). Globulin was calculated as globulin (g dL^−1^) = Total protein (g dL^−1^) − Albumin (g dL^−1^), and thereafter, the albumin/globulin ratio was also calculated.

### 2.4. Challenge Study

At the end of the feeding trial, 5 fish were randomly sampled from each replicate, resulting in a total of 15 fish from the experimental group. The fish from each replicate were then placed in separate aquaria, with three replicates maintained for each group. For the challenge study, a pathogenic strain of *A. hydrophila* was used. This particular strain of *A. hydrophila* was isolated from diseased koi carp (*Cyprinus carpio* var *koi*) and confirmed at the species level using biochemical tests, polymerase chain reaction (PCR), and matrix-assisted laser desorption ionization time-of-flight mass spectrometry (MALDI-TOF MS) [24]. The LD_90_ dose of 10^7^ cfu/fish was used for the challenge assay. The LD_90_ dose used in the present study was determined in an earlier experiment using *L. rohita* fingerlings [25]. Before the challenge, bacterial counts were adjusted using the OD_600_ values versus the viable count growth curve. For the preparation of the growth curve, 100 µL of the overnight-grown stock culture of *A. hydrophila* was inoculated into 25 mL of tryptone soy broth (TSB) (HiMedia, Thane, India) followed by incubation at 35 °C under continuous shaking at 200 rpm. At regular intervals, the absorbance of the culture was checked at an optimal density of 600 nm (OD_600_) using the microplate reader (Tecan Infinite 200 PRO, Männedorf, Switzerland. The viable counts were determined using the plate count method, as described previously [17,25]. This growth curve was used to adjust the log phase *A. hydrophila* counts to 10^8^ cfu mL^−1^. After adjusting the counts, an aliquot was centrifuged at 4500× *g* for 10 min, and the pellet was resuspended in the required volume of PBS (10 mM, pH 7.4) to obtain the final count of 10^7^ cfu mL^−1^. Each fish was intra-peritoneally injected with 100 µL of bacterial suspension (10^7^ cfu mL^−1^). A fish group that was neither fed with ginger nor infected with bacteria but injected with PBS was maintained as a negative control. The fish were observed for clinical signs (such as hemorrhages on fins and tails, feeding and swimming behavior, and mucus secretion) as well as mortality for a period of 15 days [26].

Fish from the control and challenged groups were sampled to test Koch’s postulates, i.e., to determine whether the fish injected with the bacteria displayed clinical signs and disease. The scoring of the health and disease status of the fish that were challenged was based on visual observations. We looked for visible clinical signs on the fish’s body and organs, such as lesions, discoloration, and fin rot, among others, and monitored changes in behavior, such as reduced activity levels, swimming abnormalities, loss of equilibrium, or changes in feeding patterns. To score the clinical signs, we design a scale (e.g., + to +++) where each score corresponds to a specific sign severity level. For example, No plus: No visible signs or abnormal behavior; + Less effect: mild clinical signs (e.g., slight coloration, reduced activity, or reduced feeding); ++ Moderate effect (e.g., visible lesions or significant behavior change); +++ Severe effect (e.g., extensive lesions, reddish coloration around the mouth, fin, and body, or pronounced behavioral abnormalities).

### 2.5. Statistical Analysis

The data obtained from each sampling time point (i.e., 60th or 120th day) were analyzed using one-way analysis of variance (ANOVA) to determine the effects of dietary treatments. Significant differences among the experimental groups were determined using Duncan’s multiple range tests at a significance level of *p* < 0.05. The data are expressed as mean ± standard error (SE) for each experimental group. Statistical analyses were performed using the statistical software Statistical Package for the Social Sciences (SPSS), version 20.0.

## 3. Results

### 3.1. Condition Factor

**Figure 2:**
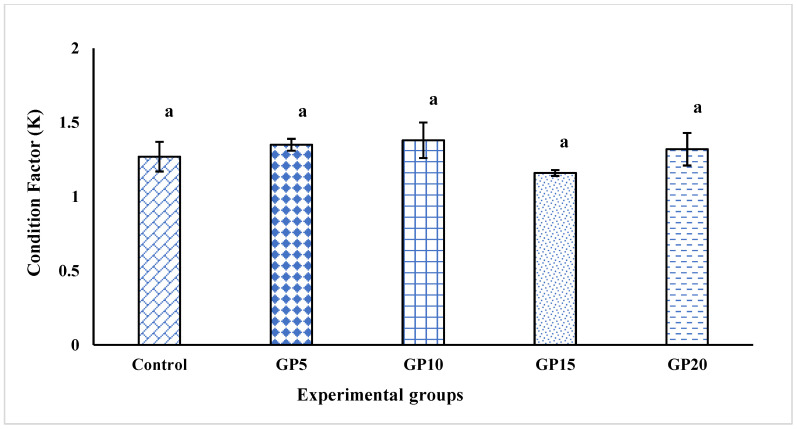
Effects of feeding experimental diets for 120 days on the condition factor (K) of *Labeo rohita*. The data are presented as mean ± standard error of the three replicates. The bars with different letters indicate significant differences (*p* < 0.05). Control (the basal diet with no GP); GP5 diet (supplemented with GP at 5 g kg^−1^ of the basal diet); GP10 (supplemented with GP at 10 g kg^−1^ of the basal diet); GP15 (supplemented with GP at 15 g kg^−1^ of the basal diet); GP20 (supplemented with GP at 20 g kg^−1^ of the basal diet).

The condition factor was not significantly different among treatments and the control group (*p* > 0.05; Figure 2).

### 3.2. Respiratory Burst Activity

**Figure 3:**
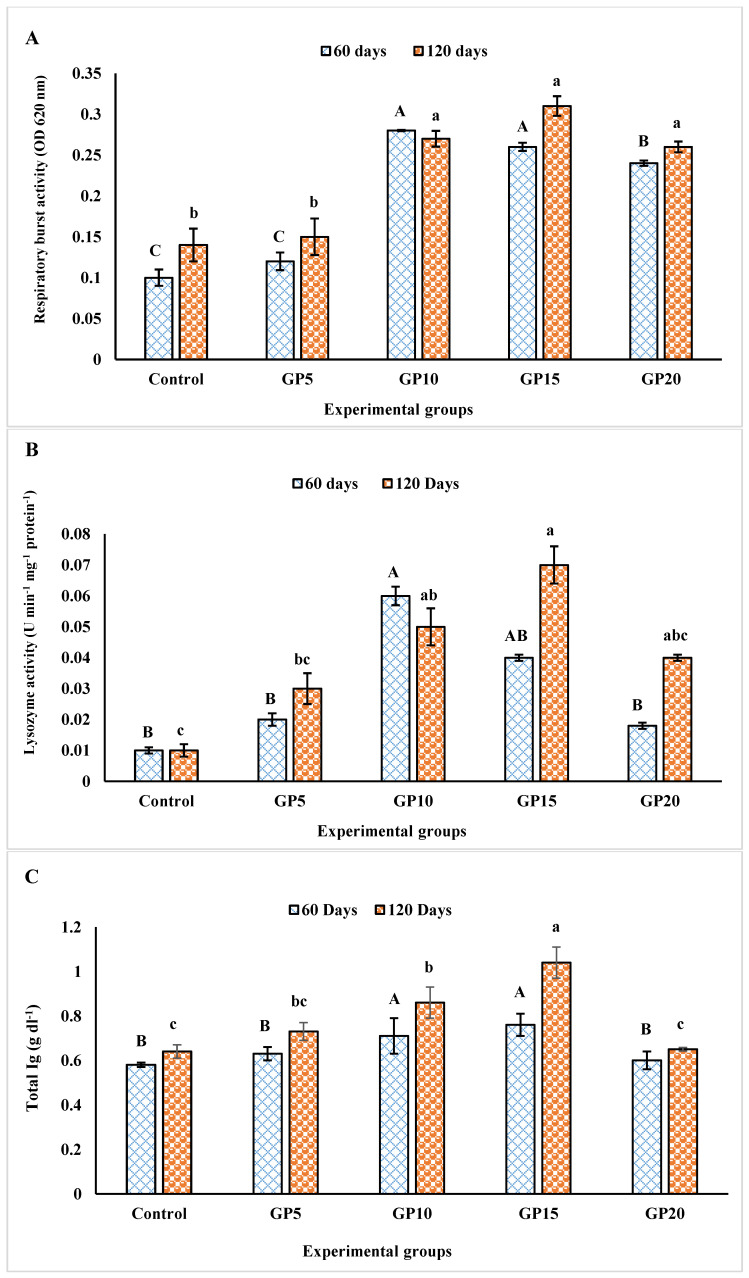
Effects of feeding experimental diets for 60 and 120 days on the (**A**) respiratory burst activity, (**B**) lysozyme activity, and (**C**) total immunoglobulin (Ig; g dL^−1^) level in the serum of the *L. rohita*. The bars with different alphabet letters (capital and small letters for day 60 and day 120 sampling points, respectively) represent significant differences between the groups (*p* < 0.05). The data are presented as mean ± standard error of the three replicates. For the experimental groups, please refer to Figure 2’s caption.

The respiratory burst activity of the fish was assessed on days 60 and 120 during the feeding period (Figure 3A). When the fish were fed with a diet containing GP at 5 g kg^−1^ of their diet, there were no effects on the respiratory burst activity of the fish on both days 60 and 120. However, when the inclusion levels of GP in the diet ranged from 10 to 20 g kg^−1^ of the diet, a significant rise in the respiratory burst activity of the fish was observed in comparison to the control group. On day 60, the GP10 group showed the highest level of activity. However, it did not differ significantly from the GP15 group. The GP20 group exhibited lower respiratory activity levels compared to the GP10 and GP15 groups. Among the groups fed GP10, GP15, and GP20 diets, no significant differences in respiratory burst activity were recorded on day 120.

### 3.3. Lysozyme Activity

The feeding of a diet supplemented with GP resulted in a significant increase in lysozyme activity on both days 60 and 120 (Figure 3B). The effect, however, was dose-dependent, with the lowest and highest doses of 5 and 20 g of GP, respectively, per kg of their diet causing no significant effect on the lysozyme activity compared to the control group on both days. The lysozyme activity was recorded at the maximum in the GP10 group on day 60 but was not significantly different from the GP15 group. Additionally, on day 60, the GP15 group also did not show a significant difference in lysozyme activity compared to the GP20 group. On day 120, the GP15 group showed the highest level of lysozyme activity (Figure 3B). However, no significant difference level was noted between the groups GP10, GP15, and GP10 on day 120.

### 3.4. Total Immunoglobulin

The total immunoglobulin level in the serum of the fish was significantly influenced by dietary GP on both days 60 and 120 (Figure 3C). The group that received the GP15 diet exhibited the highest total immunoglobulin levels on both days, followed by the group that received the GP10 diet. However, the groups that were given diets supplemented with GP at 5 and 20 g kg^−1^ of their diet did not show higher total immunoglobulin levels compared to the control group.

### 3.5. Blood Metabolic Profile

**Figure 4:**
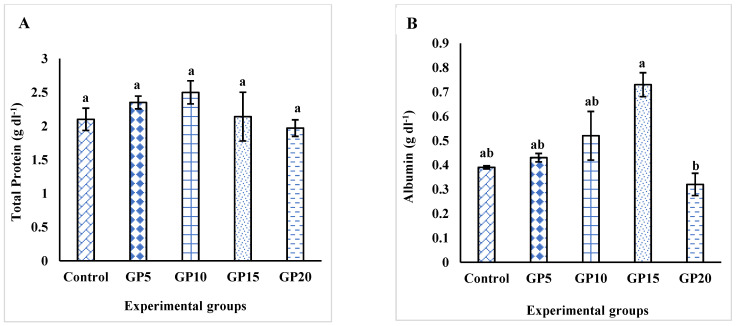

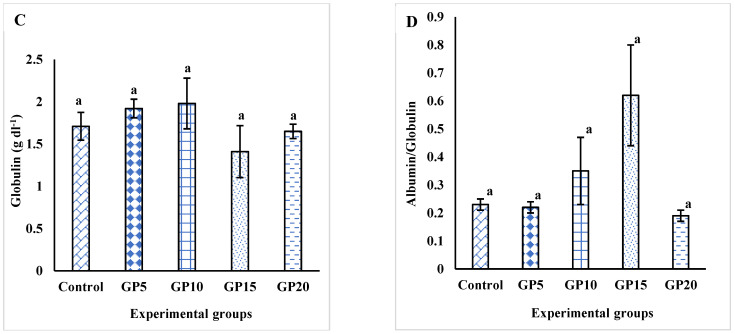
Effects of feeding the experimental diets for 120 days on the (**A**) total protein (g dL^−1^), (**B**) albumin (g dL^−1^), (**C**) globulins (g dL^−1^), and (**D**) albumin/globulin (A/G) level in the serum of *L. rohita*. The bars with different letters indicate significant differences (*p* < 0.05). The data are presented as mean ± standard error of the three replicates. For the experimental groups, please refer to Figure 2’s caption.

On day 120, the dietary GP had no significant effect on the total protein level or the globulin level, as shown in Figure 4A,C, respectively. However, albumin levels were notably higher in the GP15 group compared to all the experimental groups (Figure 4B). This increase was not significant when compared to the groups that were fed with control, GP5, or GP10 diets. In contrast, a significant difference in albumin levels was observed between the GP15 group and the GP20 group. Likewise, the GP15-fed group showed the highest albumin/globulin ratio among all the groups, approximately 2.5 times higher than the control group. However, the difference was not statistically significant.

### 3.6. Challenge Study

**Figure 5:**
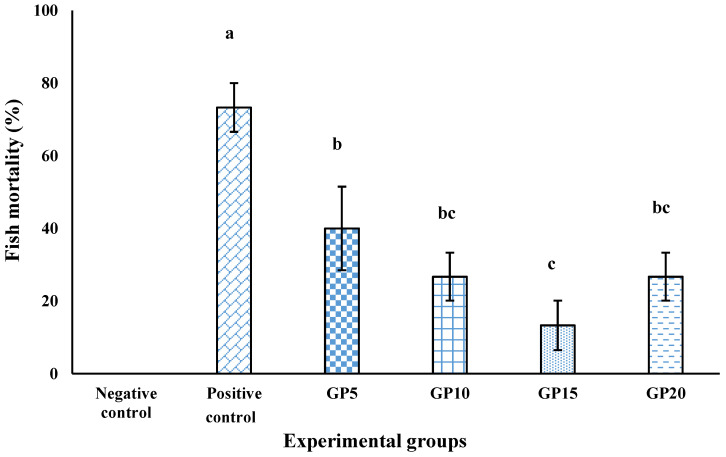
Mortality (%) of the *L. rohita* challenged with *A. hydrophila* for a period of 15 days. The *L. rohita* were fed with various experimental diets for 120 days, as indicated in Figure 2’s caption. After these 120 days, the fish were challenged with *A. hydrophila*, and the mortality was recorded over a 15-day duration. The bars with different letters indicate significant differences (*p* < 0.05). A group of fish that were not fed with GP and were not subjected to the *A. hydrophila* challenge served as the negative control. The control group, which received the control diet and was exposed to *A. hydrophila*, served as the positive control.

In the negative control group, no mortality was recorded (Figure 5). However, in the positive control group that received the same control diet but was exposed to the challenge for 15 days, a mortality of 73% was recorded. The dietary GP significantly reduced the mortality of fish caused by the bacteria. The most prominent reduction in mortality (13%) was found in the GP15 group, which was followed by the GP10 group (40%), and then the GP20 group (26%). However, there was no significant difference in mortality among the fish that were fed GP5, GP10, and GP20 diets.

**Figure 6:**
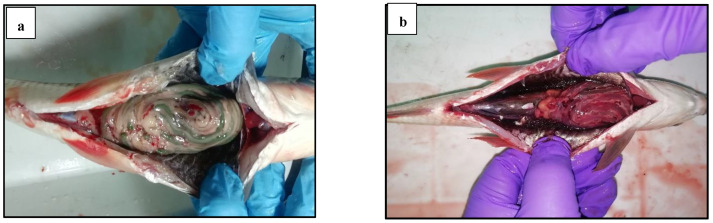

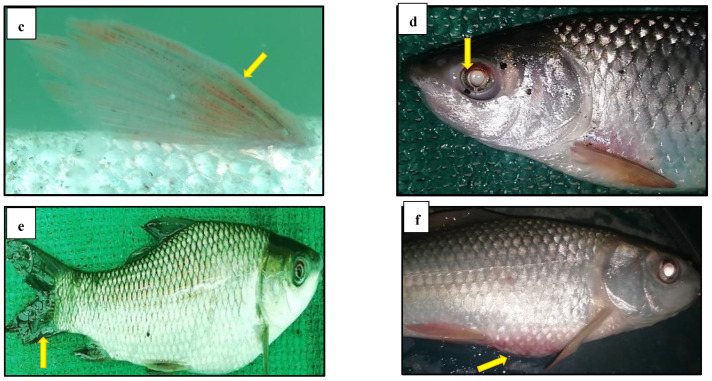
Clinical signs observed during the 15-day challenge trial involving the *L. rohita* exposed to *A. hydrophila*. For the experimental groups, please refer to Figure 2’s caption. A group of fish that were not fed with GP and were not subjected to the *A. hydrophila* challenge served as the negative control. The control group, which received the control diet and was exposed to *A. hydrophila*, served as the positive control. (**a**) Negative control: normal structure of internal organs, (**b**) positive control: inflammation of internal organs, (**c**) hemorrhages on fins, (**d**) reddish eye, (**e**) hemorrhages on the tail, and (**f**) hemorrhages on the ventral side.

Apart from monitoring mortality, the fish from the experimental groups were monitored for clinical signs (Table 1) to detect the progression and the intensity of any infection. The disease signs in the *L. rohita* challenged with *A. hydrophila* started appearing 72 h post-injection. All challenged groups, except the negative control, exhibited various clinical signs, such as loss of balance, secretions of excessive mucus on the skin, hemorrhages on certain body parts, torn and blackened fins, and skin ulcers penetrating the subcutaneous muscle (Figure 6). The most prominent disease clinical signs were observed in the positive control (Figure 6). A post-mortem examination revealed hemorrhages, inflammation, congestion, and enlargement of internal organs in the positive control, and all the groups were fed with GP and challenged with *A. hydrophila*. However, the severity of internal organ injury due to infection varied, with the positive control showing the most severe clinical signs and the GP15 group exhibiting the least (Table 1). The results of the challenge study, both for fish mortality and postmortem disease signs, were consistent with each other.

## 4. Discussion

This study was built on the findings of an earlier study by Sukumaran et al. [13], conducted on *L. rohita* under controlled laboratory conditions. It was a translational study aimed at validating those laboratory results by conducting a long-term nutritional experiment in a semi-intensive aquaculture system. The authors, Sukumaran et al. [13], used a 200-L flow-throw system to culture the *L. rohita* fingerlings of 12 g size for conducting the 60-day feeding trial with GP supplementation in the dose range of 0.2 to 1.0%. However, the feeding experiment in this study was run using 20 g fish for 120 days in 20 m^3^ cemented tanks (20,000 L), the bottom of which was covered with soil to provide natural conditions. Previously, we have demonstrated that the feeding of dietary GP at a 15 g kg^−1^ diet inclusion level led to a marked increase in the growth of the fish in comparison to the control group (for growth data, see [16]). Interestingly, the group fed the GP15 diet exhibited a marked improvement in the health condition of the fish, as manifested by a marked change in the hematological and antioxidant indices, as well as the activities of the liver enzymes. In the present study, we performed additional analyses to gain better insight into the impact of GP on the health promotion responses of *L. rohita*. We measured the fish condition factor, which serves as an indicator to assess a fish’s overall condition and well-being based on its weight and length [27,28]. Our results showed that the condition factor of the fish in all the groups was above 1.0, and the values did not differ significantly between the groups. These results suggest that GP supplementation had no adverse effect on the condition of fish and that the fish fed with GP-supplemented diets were in good condition and well-being.

To gain more insight into the health status and well-being of the fish, we measured a few selected variables associated with the defense response in fish, i.e., respiratory burst and lysozyme activities, total immunoglobulin, total serum protein, and albumin and globulin levels. Respiratory burst activity is one of the most important early defense mechanisms, as it plays a key role in pathogen eradication [29]. Therefore, the respiratory burst constitutes an important indicator of fish health status [30]. Ginger possesses a broad-spectrum activity that activates phagocytosis, which is an important component of the non-specific immune system of fish [31]. In the present study, an increase in the respiratory burst activity in the ginger-fed groups could be a result of its bioactive components, such as gingerols, shogaols, and paradols, which have been reported to induce the activity of immune molecules, increasing their resistance to bacterial diseases [32,33]. Thus, fish become more protective against infection. These results are in agreement with the findings of Dugenci et al. [11], who reported enhanced respiratory burst activity of leukocytes in rainbow trout after feeding ginger extract (1%). In another study, Talpur et al. [10] also reported a significant increase in the respiratory burst in sea bass fed ginger-supplemented diet at 3 g kg^−1^ of their feed. In our study, the highest respiratory burst activity was observed in the groups fed with GP in the dose range of 10 to 15 g kg^−1^ of their diet. In this study, we did not characterize the feed additive ginger powder for its bioactive components. However, there are previous studies that have extensively studied the bioactive constituents in ginger. These studies have consistently identified diverse chemical compounds in gingers, which include phenolic and terpene compounds, polysaccharides, and organic acids (for details, see review by Mao et al. [34]). The bioactive components in ginger are mainly gingerols, shogaols, paradols, and zingerone, among others. Moreover, there are also several terpene components in ginger, such as β-bisabolene, α-curcumene, zingiberene, α-farnesene, and β-sesquiphellandrene. These components are considered to be the main constituents of ginger essential oils [35].

Lysozyme is an important component of the innate immune system of fish and acts as the initial defense mechanism against pathogens [36]. Lysozyme is actively involved in breaking down peptidoglycan found in the bacterial cell membrane, resulting in cell lysis [37]. Previous studies have reported the effects of ginger on lysozyme activity in various fish species, such as rainbow trout [38], Asian sea bass [10], and beluga [39]. In our present study, we observed that the *L. rohita* fed diets containing GP in the dose range of 10–15 g kg^−1^ showed a significant increase in lysozyme activity compared to the control group. This suggests that GP was responsible for enhancing lysozyme activity, possibly through its biologically active components, thereby confirming its beneficial effect on the innate immunity of *L. rohita*. Plant-derived bioactive compounds have the potential to boost lysozyme activity in organisms through either an increase in the number of phagocytes secreting lysozyme or an increase in the synthesis of lysozyme per cell [38]. The extent of changes in lysozyme activities depends on the potency and type of bioactive compounds exposed to the fish [40,41]. Our study did not quantify the number of phagocytes or measure the lysozyme enzyme itself. The observed increase in lysozyme activity was likely a result of an increase in phagocyte counts, leading to the secretion of more enzymes per cell. Our findings align with the findings of previous research by Haghighi et al. [38] and Talpur et al. [10], where dietary ginger had a significant impact on serum lysozyme activity in rainbow trout *Oncorhynchus mykiss* and Asian sea bass *L. calcarifer* when included at a 1% ginger concentration. Similarly, Vahedi et al. [39] demonstrated a significant increase in lysozyme activity when ginger extract was incorporated into the diet of *Huso huso* at a 1.5% inclusion level. Likewise, Hassanin et al. [5] observed a significant rise in lysozyme activity in Nile tilapia when ginger (1%) was included in their diet compared to the control group.

Total serum protein serves as a valuable indicator of health and stress indicator in various organisms, including fish [38,42]. Total serum protein refers to the combined concentration of proteins present in the blood. It encompasses a wide range of proteins with various functions, including albumins, globulins, enzymes, hormones, and immunoglobulins. In our study, none of the tested variables, i.e., total serum protein, albumin, globulin, and albumin/globulin were significantly altered by dietary ginger. The groups fed diets supplemented with GP at 10 and 15 g kg^−1^ of their diet appeared to show higher values of albumin and albumin/globulin. However, the difference was not significantly different when compared with the control group, which could be explained by high standard errors. Our results are consistent with a previous study that had shown no positive effect of dietary ginger on the total protein and globulin content of beluga [39]. There were also earlier reports showing the effect of dietary ginger on increasing serum albumin content in tilapia and sea bass [10,12]. An improved metabolic profile in terms of serum protein, albumin, and globulin in fish generally reflects a stronger innate immune response in fish [43]. However, in our study, we did not observe a strong response in the tested variables due to feeding dietary GP. We, therefore, measured total immunoglobulin levels as a readout of the immune response. Total immunoglobulins in fish refer to the collective amount of antibodies present in the immune system of fish species. While the specific types and quantities of immunoglobulins can vary among different fish species, IgM is the most common class of antibodies found in fish [44]. Immunoglobulins are poly-reactive, showing reactivity for non-self-associated molecular patterns like LPS viral and parasitic products. It has been suggested that dietary ginger exerts its impact on the immune system in fish by modulating Ig levels [45]. In our study, increased levels of Ig were detected in the serum of fish fed diets containing 10–15 g kg^−1^ ginger before exposure to *Aeromonas hydrophila*. Our results coincide with the investigations of Sukumaran et al. [13], who observed increased total Ig levels in *L. rohita* when fed with 0.8% dietary ginger powder. Mohammadi et al. [46] further supported these findings, showing improved immune parameters in common carp fed with ginger extract, including increased erythrocyte and leukocyte counts, as well as higher levels of total immunoglobulins. The exact mechanism by which ginger powder induces immunoglobulin production in fish, including *L. rohita,* remains unknown. Such responses could likely be due to the complex interactions between the bioactive compounds in ginger and the fish’s immune system. It is likely that GP or its components may trigger certain immune cells or signaling pathways involved in immunoglobulin synthesis and secretion. However, this was pure speculation and warrants further research.

Although immune response studies offer valuable insights into the effects of feed additives, they are insufficient for a comprehensive assessment. To gain a more comprehensive understanding of the impact of feed additives on the overall health and well-being of the animals under study, it is necessary to supplement immune response data with survival results from challenge studies. Therefore, we conducted a challenge study by exposing fish that were fed with GP for 120 days to a pathogenic strain of *A. hydrophila*. The positive control group, which was fed a standard diet, experienced a mortality of over 70%. In contrast, all groups that were fed with GP demonstrated significant protection against the *Aeromonas* challenge. The highest level of protection, at around 82%, was observed in the group that had received dietary GP at an inclusion level of 15 g kg^−1^.

The increased survival rates of the fish in response to challenges with pathogenic bacteria, particularly in correlation with higher lysozyme activity and total Ig levels, clearly indicate that prolonged dietary supplementation with ginger effectively generated a protective immune response in fish against *A. hydrophila* infection. These findings align with a previous study by Talpur et al. [10] on *Lates calcarifer*, which highlighted the beneficial effects of ginger in terms of disease protection due to improved immune responses. This was evidenced by the higher survival rates of the treated groups of *L. calcarifer* after infection with *V. harveyi*. In another study, Sukumaran et al. [13] reported that *L. rohita*, when fed with 0.8% ginger powder, exhibited significantly increased survival rates after a challenge with *A. hydrophila*. Additionally, bath treatment of *Gyrodactylus turnbulli*-infected fish (*Poecilia reticulata*) using ethanolic ginger extract led to a substantial reduction in infection prevalence and intensity compared to water extract and ethanol controls [47]. The results, which demonstrate reduced mortality and lower disease incidence in ginger-fed fish, can be attributed to the presence of bioactive components in ginger, such as gingerols zingerone. These components possess multifaceted properties, including immune enhancement (as indicated by increased lysozyme and total Ig levels), as well as antioxidant, anti-inflammatory, and antibacterial properties [48,49,50]. It is also noteworthy to mention that the sample size in the challenge assay when determining survival response plays a key role in the reliability and accuracy of the results obtained. In our previous study [51], we used a sample size of 12 fish from each experimental group for the challenge study. Other research, such as Talpur et al. [10], used 15 fish per experimental group, and Hassanin et al. [5] utilized 20 fish per group for their challenge trials. To adhere to the 3Rs principles (Replacement, Reduction, and Refinement) and considering prior studies described above, we opted to use a total of 15 fish from each experimental group for our challenge study. From the subtle difference in survival response of the challenge study between the experimental groups and the correlation of the survival results with the immune response, it is evident that dietary GP generated a protective defense response in the *L. rohita* against the *A. hydrophila* challenge.

## 5. Conclusions

In conclusion, this study suggests that feeding *L. rohita* with ginger powder for 120 days can boost their immune responses and metabolic profile in their blood. This, in turn, increases their resistance to *A. hydrophila* when cultured in a semi-intensive culture system. Given the widespread availability of ginger, it is recommended to incorporate it as a natural additive into the fish feed at a dosage ranging from 10 to 15 g kg^−1^ for at least 60 days and up to 120 days to improve the overall robustness of the fish. The status of the bioactive compounds, in terms of composition and bioactivity, in the test diets was not examined in our study. The feed was prepared using a cold-pelleting process to prevent the loss of active compounds. While this process helps preserve these compounds, it may not be sufficient to assume their efficacy without proper analysis. In the future, it is essential to analyze the feed for bioactive components to determine which specific components contribute to the growth and health performances of the fish, as observed in this study. The precise molecular mechanisms through which dietary ginger powder promotes fish growth and health conditions are unclear. Various botanicals present in ginger may interact synergistically or additively to yield these beneficial impacts. More research is necessary to isolate and characterize the active compounds in ginger and to investigate how they function to enhance fish health and disease resistance.

## Figures and Tables

**Table 1:** Clinical signs observed during the 15-day challenge trial against *Aeromonas hydrophila* in *Labeo rohita*.

Days	Clinical Disease Signs	Negative Control	Positive Control	GP5	GP10	GP15	GP20
1–7	Dark fins (blackish in color)	-	+++	++	++	+	++
	Sluggish movement and reddish color around mouth	-	++	++	++	+	+
	Rejection to feed	-	+++	+++	++	+	+
	Reduced body movements and reddish abdomen	-	++	++	+	+	+
	Increased mucus on body surfaces	-	+++	+++	++	-	-
	Reddish on ventral side, lips, and eyes	-	+++	++	++	-	+
	Mortality	-	+++	++	++	+	++
8–15	Surviving fish were in good conditions but demonstrated reduced feed intake	-	+++	+++	++	+	++

Note: + Less effect; ++ Moderate effect; +++ Severe effect.

## Data Availability

Data will be made available on request.
